# Structural Determinants of Workforce Participation after Retirement in Poland

**DOI:** 10.1007/s12062-017-9213-3

**Published:** 2017-12-27

**Authors:** Olena Oleksiyenko, Danuta Życzyńska-Ciołek

**Affiliations:** Institute of Philosophy and Sociology, Polish Academy of Sciences, Nowy Świat 72, 00-330 Warsaw, Poland

**Keywords:** Workforce participation, Retirement, Structural determinants, Socio-economic status, Cumulative advantage/disadvantage approach

## Abstract

In this paper, we aim to analyse selected structural determinants of workforce participation after retirement in Poland. By *structural determinants* we mean characteristics of one’s socio-economic position that (a) result from the interplay of social conditions (mechanisms of power, differentiated access to resources) and individual agency, and (b) restrict or facilitate individuals’ choices. We conceptualise *workforce participation* as engaging in either part- or full-time paid employment despite receiving the old-age pension. Our general hypothesis is that working in older age is not only a matter of motivation or psychological traits but also a complex interplay of structural characteristics, accumulated by individuals during their life course. In the paper, we test a number of hypotheses about the role of specific components of socio-economic status (SES), i.e. occupational prestige, education, and wealth, for workforce participation among retirees. We argue that, in case of retirees, the prestige of the last job before retirement is a more reliable measure of the social position than education. Hence, we conduct a more detailed analysis of the role of occupational prestige for the chances of being employed after retirement. The analysis was based on data gathered in the years 2013–2014 within the sixth wave of the Polish Panel Survey POLPAN (www.polpan.org). We extracted a subsample of retirees from this dataset and used logistic regression to test the hypotheses described above. We found that both occupational prestige of the last job before retirement and educational attainments are strong predictors of being in paid work after retirement, however the impact of occupational prestige varies across the groups with the lowest and higher level of retirement pension. We also found that there are horizontal differences in the occupational structure of the chances for workforce participation after retirement and additionally found that being a farm owner increases the propensity to engage in economic activity after retirement. The paper contributes to the field of studies on the relationship between SES and workforce participation after retirement in line with the cumulative advantage/disadvantage theory and shows that resources that individuals have accumulated during the life course can determine their chances of working after retirement just as individual motivations or organisational characteristics do.

## Introduction

The evidence from various studies shows a growing tendency towards continued workforce participation after reaching the legal retirement age, mainly due to longer life expectancy and better health conditions of older workers (Beehr and Bennett 2015; Komp et al. 2010; Wahrendorf et al. 2016). The meaning of work for retirees and older employees is different than for younger people (Friedmann and Havighurst 1954; James and Pitt-Catsouphes 2016; Smyer and Pitt-Catsouphes 2007). The decision to continue work or to retire completely can be purely voluntary or based on financial or other economic (e.g., health insurance) reasons (Fisher et al. 2016). In this paper, we argue that the choice can be influenced by the amount of resources accumulated during the life course. We concentrate on the effects of structural factors such as the level of education, occupational prestige and wealth on workforce participation among Polish retirees.

A literature review reveals that the type of employment after reaching the retirement age can be conceptualised as *bridge employment* (Bennett et al. 2016; Cahill et al. 2006), *post-retirement employment* (Fasbender et al. 2015) or *re-entry* (Hiscott 2013), and *phased or partial retirement* (Alcover et al. 2014). According to Alcover et al. (Alcover et al. 2014), phased retirement refers to work for a reduced number of hours for the same employer, while partial retirement describes a situation when an individual moves from a long-term career to a new position. Bridge employment refers to the symbolic “bridge”, or transition from the job before retirement to complete retirement, or from the job before retirement to any paid work (part-time, full-time, temporary, self-employment) that a person might take up once they start receiving a pension (Alcover et al. 2014; Sterns and Subich 2005). The main difference between the two strategies of gradual retirement and bridge employment is that the latter is generally associated with a change of employer or switching to self-employment (Cahill et al. 2006). On the other hand, re-entry or post-retirement employment can be conceptualised both as starting a new job after a planned break in employment or an unplanned return to the labour force due to a failure in maintaining a satisfactory living standard or due to other reasons (Fasbender et al. 2015; Hiscott 2013).

While these definitions capture different patterns within the continuous process of labour force withdrawal, it is not always easy in practical terms to distinguish between the types of employment people undertake after meeting the legal criteria for retirement. To exemplify the conceptualisation problem, we look at the difference in understanding the term “post-retirement employment”. Fasbender et al. (Fasbender et al. 2015) refer to post-retirement employment as “a late career development stage”, while other researchers use the terms “post-retirement” and “bridge employment” interchangeably (Hiscott 2013) or use it to describe the pattern of employment among those who returned to paid job after retirement (Schellenberg et al. 2005).

In this paper, we focus on any type of paid employment a person may have after meeting the legal criteria for retirement and moving to a pension. Due to data limitations, we are not able to distinguish between the different forms, working hours, change of employer, gaps in employment (re-entry), or continued post-retirement employment. However, we are aware that the determinants of workforce participation can vary across types described in the literature mentioned above. We use the term “workforce participation after retirement” with the aim to analyse structural determinants of any form of paid jobs that retirees engage in, despite receiving retirement benefits.

To understand the context of the study, we will briefly describe the tendencies in employment patterns among Polish employees, and the changing social context related to the processes of social, economic and political transformation in Poland after 1989. In Western Europe and the USA, retirement is no longer perceived as a single event indicating the end of full-time career and withdrawal from the labour market but, rather, as a multi-stage decision process regarding employment patterns in older age (Beehr 1986; Beehr and Bennett 2007; Shultz and Wang 2011). In Poland, working in old age is not a common phenomenon yet. The role of an employee or business owner is still quite new for older Poles. However, considering the increasing life expectancy, the improving health situation of older people and the recent demographic changes, we expect that an increasing number of Poles will carefully consider the dilemma “to retire or not to retire?” in the coming years. And if the answer is affirmative, then they will need to decide how to manage the process of workforce withdrawal.

## Retirement in Poland: Legal and Socio-Economic Background

Most Poles still perceive retirement as a single decision rather than a process. Governmental statistics indicate that retirement in Poland rarely occurs gradually. For example, a study conducted in 2012 showed that „[i]n the population of the employed (irrespective from receiving social benefits^1^) or economically inactive and receiving social benefits aged 50–69 years only 8.7% declared that they had reduced the number of working hours in the move towards final transition into retirement. Among persons employed and receiving social benefits the percentage share amounts to 24.1%; in most cases, reduction in the number of working hours took place after receiving the first old-age pensions” (GUS [Central Statistical Office of Poland] 2013, p. 84). Polish researchers find evidence to support these observations. For example, Zientara (Zientara 2014) highlights the need for more flexible workplace arrangements that would help older employees to move more gradually from work to retirement. This situation is reflected in terminology used in this paper. We write about “workforce participation after retirement” bearing in mind the job an individual performs after (s)he was granted retirement benefits.

The vast majority of people who work in Poland under legal agreements are covered by the compulsory pension insurance scheme. Individuals gain pension entitlement upon reaching the statutory retirement age. Apart from a few exceptions, Poles working outside agriculture and born before 1 January 1949 are entitled to receive an old-age pension if they can document that they had paid pension contributions during the required period (20 years for women and 25 for men). Individuals born after this date must document the period of paying the pension contributions only if their contributions to the insurance system have not been sufficient to make them eligible for the minimum pension.^2^ If their contributions have been high enough, they can retire after reaching the retirement age without any additional conditions. Farmers are covered by a separate pension system and, essentially, they must reach the retirement age and document 25 years of insurance to become entitled to old-age pension.

During the last 30 years, several significant reforms of the Polish old-age pension scheme were conducted. For many years before 1991, the only governmental institution responsible for granting old-age pensions was the Social Insurance Institution (ZUS).^3^ In 1991, the Agricultural Social Insurance Fund (KRUS) was established to implement the new agricultural social insurance system for farmers. In 1999, a major reform of the pension system was introduced. The new law introduced, among others, a compulsory “second pillar” for individuals born after 1948 and insured in ZUS. In 2014, participation in the “second pillar” became voluntary. In 2013, a reform equalising the retirement age for men and women and raising it to 67 years (previously: 60 years for women and 65 for men) was introduced. In December 2016, the new government decided to restore the previous retirement age, starting from October 2017. The number and frequency of changes in the pension system has undermined the public trust in the system and has led to a negative perception of the system’s stability (Krzyżanowska 2011).

The Polish labour market is not friendly for older employees. For example, Turek (Turek 2015) analysed data from a large Polish study of employers, Human Capital Balance 2010–2013, and found that a specified age requirement is a common feature during the recruitment process: 79% of employers seeking an employee declared clear age preferences. In this group, candidates aged 25–40 turned out to be most desirable. There was a rapid decrease in declared willingness to employ persons over 40: for example, only 27% of employers were eager to offer a job to someone aged 50. The Polish Government Plenipotentiary for Civil Society and Equal Treatment indicates several other domains of age-based discrimination on the Polish labour market, unrelated to recruitment, namely: limited access to professional training, dismissal when the person has reached the retirement age, or mobbing due to older age.^4^ There is no clear evidence that ageism is the major factor restraining occupational activity of older Poles. Nevertheless, the employment rate in the age group 55–64 is significantly lower than in other EU or OECD countries. According to the latest available official data compiled by OECD (OECD 2017a), the employment rate in this group in Poland reached 44.3% (as for 2015), while the mean for all OECD countries was 58.1%. Poland ranked 30th in the 2016 edition of the Golden Age Index ranking, published by PwC and covering 34 OECD countries (PwC 2016). The Golden Age Index takes into account a broad range of indicators capturing the participation of people aged 55+ in employment and training. Unfavourable labour market circumstances may push some older employees to retire in order to avoid unemployment. This tendency is sensitive to changes in the economic situation connected with historical events. In the beginning of 1990s, different forms of early retirement, including bridging pensions, were very popular among people at a risk of collective dismissals. Moreover, some people decided to retire because they were afraid of certain expected unfavourable changes in retirement regulations.

Another “piece of the puzzle” refers to the amount of old age pensions. According to the Central Statistical Office of Poland (GUS), in January 2017 the average monthly gross retirement benefit from non-agricultural insurance system was equal PLN 2220.11 (Polish zlotys), whereas farmers insured in KRUS received PLN 1192.30 on average (GUS [Central Statistical Office of Poland] 2017). At the same time, the statutory minimum monthly wage was PLN 2000. In 2014, the net pension replacement rate^5^ in Poland was 53%, compared with the OECD average of 63% (OECD 2017b). The difference of 10 percentage points could be considered relatively low, but there are three issues worth pointing out. Firstly, there are significant gender differences with regard to the amount of retirement benefits. As for old age pensions paid by ZUS, the median for men was equal to PLN 2301.99 in March 2016, while the corresponding value for women (who constitute about 60% of Polish retirees) was PLN 1590.68 (ZUS [Social Insurance Institution] 2016). Secondly, according to the forecasts for the following years, the situation will considerably worsen due to population ageing and unsustainability of the pension system. Thirdly, there are many exceptions in the Polish pension regulations and, therefore, some individuals have the right to retire earlier than others, or, for example, to receive early retirement benefits. For them, a shorter working period means lower retirement benefits. It is also worth mentioning that in comparison with other European nations Poles have relatively low private savings and rarely save money for retirement (World Bank 2014).

## Determinants of Workforce Participation after Retirement: Research Hypothesis

Despite certain terminological inconsistency, the literature on factors stimulating workforce participation after retirement is rich and growing. The decision to remain active professionally can be analysed in terms of older workers’ motivations to remain on the labour market. According to Mor-Barak (Mor-Barak 1995), the factors which determine these decisions can be divided into four main groups: social (receiving status and maintain social bonds), personal (self-esteem and life satisfaction), generative (passing knowledge on to younger generations), and financial (maintaining pre-retirement income). Continuity theory assumes that retirees remain the same people as before, i.e., their attitudes and temperament do not change and they would rather engage in the same activities as previously, e.g. working in the same or similar jobs (Atchley 1989). A retiree might experience rolelessness due to the absence of their usual daily timetable (Kim and Feldman 2000). Therefore, some of them may choose to continue careers to reduce the level of stress associated with the changing roles. A comprehensive review of the literature on psychological conceptualisations and models for understanding retirement can be found Wang and Shi (Wang and Shi 2014).

Motivations, however, do not emerge irrespectively of the social structure and each aspect of work can have a different meaning for the individual concerned, depending on gender, ethnic group or socio-economic status (e.g., Hughes and O’Rand 2004). The combination of psychological motivation and structural determinants can be found in the classification of mature workers by Nakai et al. (Nakai et al. 2011). They found that the first group of such workers, called *satisficers*, consists primarily of married individuals and males who choose to work for economic and family reasons, the second group, *free agents*, includes mostly unmarried people and females who choose to work for personal reasons; whereas females and individuals with low qualifications seeking employment for various reasons are classified as *maximizers*. This means that not only age but also gender and marital status need to be controlled.

Considering the role of social inequalities, one way to look at the employment choices in older age is through the lens of the cumulative advantage/disadvantage theory which perceives inequalities among old people as a consequence of the unequal allocation of resources such as education or employment during the life course (Dannefer 2003). According to Dannefer (Dannefer 2003), the effect of advantage/disadvantage accumulation goes along the lines of popular sayings, such as “success breeds success” or “rich get richer, the poor get poorer”. This approach can be also situated within the frame of the multilevel model of retirement, described by Wang and Shi (Wang and Shi 2014). In our paper, we adopt this theoretical frame, focusing on the microlevel factors influencing the workforce participation after retirement. We concentrate on individual attributes such as the level of education, prestige of the pre-retirement job, amount of retirement benefits, health status and selected demographic variables.

Previous empirical studies have shown that the cumulative advantage/disadvantage approach can be relevant for studies of employment in older age. For instance, according to Komp et al. (Komp et al. 2010), the propensity to engage in paid employment is higher among better-educated males and females who achieved a higher socio-economic status (SES). The authors distinguish between three different components of SES: wealth, education and occupational prestige. They stress that those components might, but do not have to, be associated with each other. Wealth is not necessarily only income-based, while income is not equally distributed among men and women, and the link between prestige and income can be blurred. Therefore, different components of SES should be analysed separately to show the real effect of each factor (Komp et al. 2010). We follow the same approach in our study bearing in mind the specificity of post-communist countries, Poland being one of them.

Education, the first of SES components that we focus on, is an unequally allocated resource. There is a clear evidence that the attained level of education is associated with higher employment rates before retirement (e.g. Aud et al. 2012). The odds of being in paid job for the retirees also increase with their level of education (Shultz 2003; Griffin and Hesketh 2008; Komp et al. 2010). According to Griffin and Hesketh (2008), better educated older respondents might be driven by the generative motivation. Their chances of finding employment are higher due to accumulated social capital and skills and they are generally perceived as more attractive employees than those with a lower level of education (Griffin and Hesketh 2008; Komp et al. 2010). Another explanation for the higher chances of being employed as a retiree is that individuals who continued their education for a longer period of time enter workforce later and continue working for a longer period of time (Komp et al. 2010). In view of these results from previous studies, we have formulated the first hypothesis about the impact of education on chances of being in paid employment after retirement:

1. *Better educated respondents (with secondary or higher education) are more likely to be employed after retirement than those with a lower level of education (below secondary).*

Another aspect of the socio-economic status, i.e. occupational prestige, is also closely related to the issue of workforce participation after retirement. Based on earlier finding from international (e.g. Kahl and Davis 1955) and Polish studies, Domański et al. (Domański et al. 2012) argue that occupational prestige can be treated as a valid and reliable indicator of the social position. The scale of occupational prestige can be perceived as a universal measure of the position in the social structure. Various scales of occupational prestige implemented in Poland show a high degree of consistency across time, and despite the historical changes occurring in Poland, the highest occupational prestige was always associated with occupations that require tertiary education (e.g., university professor or medical doctor), occupations characterised by high salaries (e.g. director of a company, business owner) or occupations which ensure the social order and facilitate socialisation (e.g., judge, teacher). Scales of occupational prestige exhibit high level of consistency across social classes and countries, hence it can be said that occupational prestige is perceived in similar way irrespective of individual characteristics, cultural differences and changes over time (see: Domański et al. 2010; Treiman 1977).

Some studies suggest that job satisfaction is associated with the ascribed job prestige (Smith 2007). According to Duemmler and Caprani (Duemmler and Caprani 2016): “individuals in high-status occupations can draw on collectively recognised ideologies to legitimate their status, those in low-prestige jobs have to create and maintain such ideologies in everyday life” (Duemmler and Caprani 2016, p.2). Based on these findings, we can assume that retirees who were in more prestigious occupations may have a positive attitude towards their job and exhibit a higher interest in continued work (Komp et al. 2010). What is also worth noting is that high occupational prestige has positive psychological aspects, such as high esteem, respect and sense of integration it provides for the individual (Domański 2012). In Mor-Barak’s (Mor-Barak 1995) terms, occupational prestige can serve as a social and partially personal motivation to remain active professionally. But if we consider occupational prestige in relation to wealth, high occupational prestige is not always associated with a high income, therefore in some cases high prestige can be perceived as a compensation for low income and vice versa (Domański 2012). Hence, one can hypothesise that the impact of occupational prestige on decisions to continue working after retirement can be different for individuals with high and low level of income and retirement benefits.

Based on these findings, we have formulated the following hypothesis related to the impact of occupational prestige on workforce participation after retirement:

H2: *The probability of workforce participation after retirement increases alongside with the increase in prestige associated with the last job before retirement*.

Next, we consider occupational prestige in the context of financial situation of retirees. Working after retirement can, but do not have to, be caused by economic pressure only (Kim and Feldman 2000). It is worth mentioning that, according to Wang and Shultz (2010), who analysed many studies examining the reasons for retiring, financial motivation is not always the leading factor to determine an individual’s decision to retire or keep working. Hence, people may be motivated not to withdraw from the labour market due to their attachment to the career or organisation (see the notion of career bridge employment, Wang et al. 2009). As we argued above, based on many empirical studies, a positive attitude towards job and career is proved to be associated with occupational prestige. Therefore, we hypothesise that the prestige of the pre-retirement job interacts with the level of retirement benefits and modulates the impact of one’s financial status on the probability of workforce participation after retirement. In case of non-prestigious pre-retirement jobs we expect that respondents who have low retirement benefits are more likely to work after retirement than those who are better off, as the former are strongly motivated economically to do so (i.e., they have to find an additional source of income). On the other hand, we hypothesise that in the case of more prestigious pre-retirement jobs the impact of financial factors on the probability of workforce participation after retirement diminishes because even respondents who receive high retirement pensions are likely to work, driven by a variety of non-structural motivations, e.g., job satisfaction.^6^

Our hypotheses with regard to the interplay of prestige and wealth are as follows:

H3: *The effect of the occupational prestige is different for groups of respondents with low and high retirement pensions, namely:*

- *H3a: Retirees who have low pensions and were previously performing jobs with a low occupational prestige are more likely to work after retirement than retirees with higher pension benefits working previously in “non-prestigious” occupations;*
- *H3b: With the increase in prestige scores, the difference in the probability of workforce participation between respondents in the lowest quintile of the retirement benefits and respondents who are better off diminishes. In the most prestigious occupations, the chances of continued workforce participation for respondents falling into the lowest quintile are just as high as for those in the most prestigious occupations, but falling into quintiles other than the lowest one.*

One needs to remember that farmers in Poland are covered by a separate pension scheme (KRUS) and they receive, on average, significantly lower retirement pensions than Poles working outside agriculture. On the other hand, Poland’s agriculture has its special characteristics. There are many individual, relatively small farms in Poland (the average size of a farm in 2016 was 10.56 ha^7^), and over a half of them are mainly or exclusively subsistence farms, this reducing their expenses on food and family maintenance (Jabłońska-Urbaniak 2012). Moreover, older farmers who have retired still “live in their workplace” and often work together with their children, so they are driven by different factors than “non-agricultural” workers. As horizontal differences in the occupational structure might blur our interpretation, we decided to conduct an additional analysis and add a dichotomous variable indicating whether the respondent’s last job before retirement was in farming or not.

H4: *Being a farm owner increases the chances of workforce participation after retirement, controlling for other structural determinants.*

It is important to stress that all the structural determinants are interrelated and the possible effect on the workforce participation among retirees must be analysed with respect to all the factors. For instance, females are more likely than males to engage in paid employment (e.g. bridge employment) in older age. Females are less likely to accumulate financial resources while being professionally active due to their temporary exits from workforce and caregiving duties (Kim 2009; Quinn and Kozy 1996). The health status of older workers is another important aspect of labour force participation after retirement, as acknowledged in literature (e.g. Davis 2003; Kim and Feldman 2000) and it is associated with status characteristics (Dannefer 2003). Health is also related to age and, therefore, older retirees are less likely to work than their younger counterparts (Davis 2003; Kim and Feldman 2000).

## Data and Method

We have used the data from the sixth wave of the Polish Panel Survey POLPAN, conducted in 2013–2014 (hereafter: POLPAN 2013). POLPAN is a panel study of the Polish society, initiated in 1987–1988 and continued in five-year intervals. The respondents selected for the study in 1987 constituted a random sample, representative for the Polish population aged 21–65. To ensure an adequate age balance, additional subsamples involving young cohorts have been supplemented since 1998. The sixth wave of the survey covered 2780 respondents aged 21–92, with different histories of participation in the project.^8^

The study is based on face-to-face interviews. The questionnaire contains a wide range of questions regarding the course of the respondent’s career, decisions on education, views on the role of the state and economic transformations, political behaviours and attitudes, physical and psychological well-being, household composition, the standard of living and many other issues. In 2013–2014, an abbreviated mail questionnaire was also used for 199 respondents who were contacted after a long break and did not agree to an interviewer’s visit. More information on the scope, methodology and theoretical background of the project can be found in (Słomczyński et al. 2015) or at polpan.org.

For our analysis, we have selected a subsample of retirees who had been interviewed face-to-face using the full, standardiaed questionnaire. Respondents have been classified as retirees if they declared receiving retirement benefits in the last round of POLPAN.^9^

Analysis of the POLPAN survey conducted in 2013–2014 shows that only 22% of the respondents declared they wished to continue working after retirement when entitled to receive the old-age pension. According to respondents’ statements, the most common reasons for non-working despite the declared willingness were lack of job offers, poor health and family care obligations.

Table 1 presents the descriptive statistics of our subsample. The subsample consists of 787 retirees; 11.3% of the respondents declared being in paid employment at the time of the study.^10^ The age of the respondents varies from the early retirees (age 49) to 91 years of age, with the mean age of the sample equal to 70 and median equal to 69. Roughly a half of the sample (48.4%) declared having secondary, post-secondary or tertiary education. Female respondents prevail in the subsample (62.1%). Married respondents and those living with a partner constitute 67.2% of the subsample.

**Table 1:** Basic socio-demographic characteristics of respondents

Basic socio-demographic characteristics of respondents	Frequencies/descriptive statistics
Age in years	Min = 49
	Max = 91
	Mean = 70.4
	Median = 69.0
	Standard deviation = 7.9
Male	Male – 37.9%
	Female – 62.1%
Education	Secondary or higher – 48.4%
	Elementary or basic vocational – 51.5%
Marital status	Married or cohabiting – 67.2%
	Other situation – 32.8%
Farmer	Yes – 13.7%
	No – 86.3%

*Source: POLPAN 2013*

## Dependent and Independent Variables

We have defined the dependent variable, *working after retirement*, as having any type of paid work while receiving an old-age pension. The variable is dichotomous, where 1 = respondent is in paid work, 0 = otherwise.

The independent variables are: three components of SES, other socio-demographic characteristics which proved to be important predictors of the retirement in older age, and being a farm owner before retirement.

*The occupational prestige of the last job* – the variable concerns the respondent’s last job before retirement, i.e., before they started to receive an old-age pension. We use the 2009 Scale of Occupational Prestige (Domański et al. 2009).^11^ The variable is measured on a ratio scale. The scale of prestige is highly correlated with other scales used in the study.^12^ In our subsample, occupational prestige ranges from 1.4 to 91.0, with the mean value of 35.5 and median at 28.9 points, the standard deviation is 22.3.

*Low retirement benefits* – a poverty indicator; a dichotomous variable measuring wealth aspect of the socio-economic status. It was generated by dividing our subsample into quintiles based on the declared amount of retirement pension, and then aggregating quintiles 2, 3, 4 and 5. On this scale, “1” means that the respondent belongs to the lowest quintile and “0” means otherwise. In fact, our quintiles were not exactly the same size because sometimes many observations were situated at the border value. Thus, our lowest quintile includes 18.1% of the subsample (taking into account valid observations only). The idea to divide the respondents into two groups, i.e. the lowest quintile and others, was theoretically motivated. It was not our intention to analyse changes in workforce participation associated with a gradual increase in the amount of retirement benefits, but to account for the situation of retirees in the lowest quintile of the retirement benefits, i.e., poverty triggered by retirement. Hence, we wanted to distinguish the group of individuals whose retirement benefits are really low and who are faced with serious financial difficulties. The cutting point of the first quintile (PLN 999) is slightly lower than the subsistence minimum for one person (households of persons above retirement age – single-member household) that was equal to PLN 1070.65 [ca. EUR 250] in 2014.

*Education secondary or above* – a dichotomous variable indicating whether the respondent completed secondary or a higher level of education (*Education secondary or above* = 1), even if (s)he did not approach the final exam, which is not an integral part of secondary school education in Poland. “0” means that the respondent has elementary or basic vocational education or no formal education at all. The original variable was nominal and included specific educational qualifications obtained by the respondent. We decided to create the dichotomous variable distinguishing between the secondary education or above and other educational attainments, since, in our perception, due to reforms in the Polish school system these educational levels are comparable between cohorts born in different time periods and schooled in different educational systems. The issue of changes in the Polish school system, as well as the problem of cross-national comparability of education gained within it is discussed by Sawiński (2012).

*Poor physical ability NHP* is an indicator of the physical health status. The POLPAN questionnaire contains the Nottingham Health Profile, a tool designed to assess six dimensions of health (Hunt et al. 1981; Hunt et al. 1985; McDowell 2006). We use the scale called “physical ability”. It consists of eight yes/no statements, such as “I find it hard to bend”, “I have trouble getting up and down stairs and steps”, “I find it hard to reach for things”. “I find it hard to stand for long (e.g., at the kitchen sink, waiting in a line)”. Answers are weighted, and the final values fall within the range from 0 to 100. Higher values indicate deteriorated physical health while “0” denotes no problems of this kind. In our subsample, the median assessment is 11.2, the mean is 22.7, and the standard deviation is 25.9.

*Self-assessment of health* – a dichotomous variable, based on the respondent’s answer to the following question: “How do you evaluate the condition of your physical health in comparison with the physical health of the majority of people at your age? Is the condition of your physical health: (1) much better than the health of other people at your age, (2) somewhat better, (3) somewhat worse, or (4) much worse than the health of other people at your age?”. An additional answer, to be used by an interviewer if applicable, was: “(5) about the same”. We have assigned value “1” to all respondents who had chosen answers 1 or 2, and “0” to those who have chosen 3, 4 or 5. Our intention was to distinguish the group of respondents whose relative self-assessment of health was explicitly positive, and who clearly stated that their health was better than the health of other people at their age. In our subsample, 48.2% of respondents assessed their health in such way.

We decided to include two health-related variables since they measure different concepts. The first variable measures specific physical limitations in daily activities, while the second one prompts the respondents to assess their health status in a relative manner, i.e. in comparison to perceived health of other people of the same age. In our subsample, the correlation between these variables is low (−0.29).

*Married or cohabiting –* a dichotomous variable referring to the respondent’s marital status (1 = married or cohabiting, 0 = otherwise).

*Farmer* – a dichotomous variable indicating whether a respondent’s last job before retirement was “a farm owner” (*Farmer* = 1, otherwise – 0). To create this variable, we have used a division into 14 socio-occupational groups (Domański et al. 2009).

Two additional independent variables are: gender (0 = female; 1 = male) and age in years (as at the time of the interview).

## Results

Educational attainment and occupational prestige are highly correlated in our subsample of the retirees (*r* = 0.71).^13^ Therefore, in the first step we test two independent models about the predictive power of these two variables for chances of being in paid employment after retirement. Table 2 shows the multiple logistic regression results for Model 1 (with educational attainment as the main predictor out of SES components, but without occupational prestige) and for Model 2 (with occupational prestige as the main SES-related predictor, but without education as the explanatory variable). Model 1 shows support for H1, retirees who completed the secondary or tertiary education comparing to those with elementary and basic vocational are more likely to continue workforce participation after retirement. Model 2 shows support for hypothesis H2about the increased chances of being in paid employment alongside with the increase in the occupational prestige of the last job.

**Table 2:** Results of multiple logistic regression. Dependent variable: being in paid employment after retirement. Comparison of Model 1 and Model 2

	Model 1			Model 2		
Independent variables	B	S.E.	OR	B	S.E.	OR
Education (secondary and above)	0.534*	0.273	1.706	–	–	–
Occupational prestige of the last job	–	–	–	0.017**	0.006	1.017
Low retirement benefits	0.665	0.348	1.944	0.842*	0.358	2.320
Male	0.284	0.279	1.329	0.244	0.277	1.276
Age in years	−0.119***	0.022	0.889	−0.112***	0.022	0.894
Married or cohabiting	−0.130	0.300	0.878	−0.839	0.305	0.920
Poor physical ability NHP	−0.013	0.008	0.987	−0.015*	0.008	0.985
Self-assessment of health	0.649*	0.275	1.914	0.623*	0.275	1.864
Pseudo R2	0.1411			0.1370		
Sample size	n = 723			n = 712		

* p < 0.05, ** *p* < 0.01, *** *p* < 0.001

*Source: POLPAN 2013*

Looking at Models 1 and 2, we cannot assess whether H3 is supported or not. It is only possible to state that Model 2 shows a significant and positive relationship between low retirement benefits and probability of being in paid work after retirement, while there is no such relationship in Model 1.

Due to the high positive correlation between educational attainments and occupational prestige, these two variables were not combined into one model. In the next steps, we focus on occupational prestige and do not include education into the set of independent variables. The basic reason is the fact that H3, which needs more in-depth examination, refers to occupational prestige and low level of retirement benefits, and does not refer to education. Moreover, we assume that occupational prestige is a more relevant measure of the social position for retirees. While education is a strong predictor of occupational career trajectories, yet the occupational prestige of the last job is, in our opinion, a more precise indicator of occupational history and, as such, may play a greater role for people who have met the legal criteria for retirement when it comes to their chances of being employed.

To investigate the issue of status inconsistency, i.e. the impact of occupational prestige on the odds of being employed, for the respondents with high and low retirement benefits (H3a, H3b), in Model 3 (Table 3) we firstly added the interaction term of the occupational prestige and low retirement benefits to Model 2. All predictors from Model 2 are significant in Model 3. The added interaction term is not statistically significant but, according to Brambor et al. (2006), the coefficients and significance of the interaction term can be interpreted only together with the product components of the interaction. The fact that the interaction is insignificant does not mean that it cannot operate in a meaningful way. Since poverty indicator is correlated with other independent variables, the interaction effect was additionally tested for differences in the prestige parameters in two groups: “the lowest retirement benefits” and “other than lowest retirement benefits”. The null hypothesis about the lack of differences was rejected since z > 1.96 (results are significant at *p* < 0.05) for the B coefficients of the prestige effect. Based on these findings, we claim that the graphic representation of interaction is an appropriate tool for interpreting it.

**Table 3:** Results of multiple logistic regression. Dependent variable: being in paid employment after retirement. Model 3

	Model 3		
Independent variables	B	S.E.	OR
Occupational prestige of the last job	0.020**	0.007	1.020
Low retirement benefits	1.407*	0.595	4.082
Interaction term prestige x low retirement benefits	−0.021	0.018	0.979
Male	0.239	0.277	1.270
Age in years (2013)	−0.114***	0.022	0.893
Married or cohabiting	−0.044	0.307	0.957
Poor physical ability NHP	−0.016*	0.008	0.984
Self-assessment of health	0.617*	0.276	1.854
Pseudo R2	0.1398		
Sample size	N = 712		

* p < 0.05, ** p < 0.01, *** p < 0.001

*Source:POLPAN 2013*

**Fig. 1:**
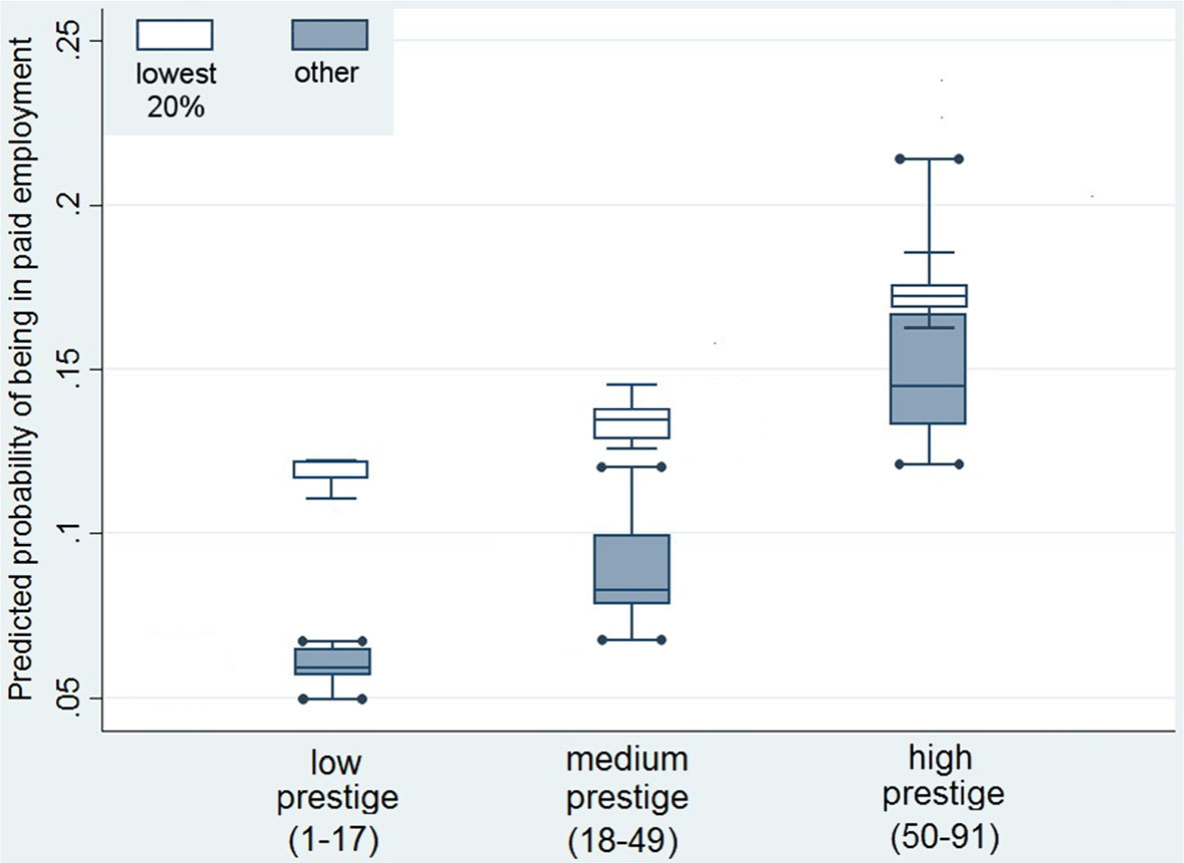
Predicted probability of being in paid employment after retirement for the respondents in the bottom quintile of the retirement benefits (“lowest 20%”) and respondents in higher quintiles (“other”). *Source: POLPAN 2013*

Secondly, we graphically predicted the probability of workforce participation after retirement for two groups: respondents who fall within the lowest 20% of the pension scale and those who are not in the lowest 20%. Figure 1 shows that for respondents receiving the lowest benefits, the probability of working after retirement is generally higher than for those in other than the lowest quintile. In the least prestigious occupations, the difference between these two groups is most visible: respondents who have low retirement benefits and non-prestigious pre-retirement jobs are much more likely to work after retirement than those who had non-prestigious pre-retirement jobs but receive higher old-age pensions. The differences are less visible in the medium section of the prestige scale, but still the poorest respondents show a higher propensity to continue working after retirement. In the most prestigious occupations, the probability to remain professionally active is only slightly higher for the respondents who receive retirement benefits falling into the lowest 20% than for respondents who have higher benefits.

The results support H3a: Retirees who have low pensions and were previously performing jobs with low occupational prestige are more likely to work after retirement than retirees with higher pension benefits working previously in “non-prestigious” occupations. This discrepancy decreases with the increase in prestige, which is consistent with H3b. The results suggest that occupational prestige can indeed serve as compensation for low income but only for the most prestigious occupations. As we mentioned above, retired respondents who are better off financially are probably driven by generative motivation to continue working in prestigious jobs, while for the respondents whose old pension is low and who are motivated mainly by financial reasons, the esteem of their job can serve as an additional motivation to persist in the workforce.

To test the additional hypothesis H4 about horizontal differences in the occupational structure, we added variable *Farmer* to Model 2. The results (Model 4) are presented in Table 4.

**Table 4:** Results of the multiple logistic regression. Dependent variable: being in paid employment after retirement. Model 4 with *Farmer* as a control variable

	Model 4		
Independent variables	B	S.E.	OR
Occupational prestige of the last job	0.022**	0.007	1.022
Low retirement benefits	0.428	0.403	1.534
Male	0.217	0.277	1.243
Age in years	−0.118***	0.022	0.889
Married or cohabiting	−0.035	0.309	0.966
Poor physical ability NHP	−0.015*	0.008	0.985
Self-assessment of health	0.662*	0.278	1.939
Farmer	1.189**	0.460	3.282
Pseudo R2	0,1495		
Sample size	***N*** **=** 711		

* *p* < .05, ** *p* < .01, *** *p* < .001

*Source: POLPAN 2013*

The results show that being a farmer is a significant predictor of workforce participation after retirement in Poland, controlling for other independent variables included to the model. If this control variable is added, this also improves the fit of the model comparing to Model 3. Hence, Hypothesis 4 is confirmed by the data. Occupational prestige remained significant in Model 4 but low retirement benefits lose significance when horizontal differences are controlled for. The topic of differences between the probability of being in paid employment among farmers and non-farmers is not a key aspect of research dealing with the structural determinants of workforce participation after retirement. However, these findings add an additional layer to the analysis of structural determinants and pave the way for further investigations of the horizontal differences within the occupational structure.

## Conclusions and Discussion

Active aging strategies might, but do not have to, be associated only with individual motivations since there is clear evidence that structural determinants can influence the probability of choosing to continue one’s workforce participation after retirement. The chances of employment among the older employees should not be studied only in the context of the demographic panic (Palska 2004) about the future of the rapidly aging societies, but also in the context of persisting and growing inequalities which greatly impact the length and quality of occupational careers of the future retirees.

To summarise the results of our analysis, we find that H1 about the role of educational attainment for the chances of being employed in older age is supported. The results show complete support for H2 concerning the role of occupational prestige for workforce participation after retirement. H3, H3a and H3b concerning the role of wealth are also supported. We found that the effect of occupational prestige varies, depending on the position on the scale of retirement benefits (lowest 20% or not). As for non-prestigious pre-retirement occupations, the poorest respondents are more likely to work after retirement than retirees with higher pension benefits. We also found that the chances of working after retirement for the most prestigious pre-retirement occupations are relatively high irrespective of the level of retirement benefits received.

The auxiliary hypotheses H4 is also confirmed: working before retirement in an agricultural sector increases the chances of workforce participation after retirement. This result calls for further analysis. Our findings suggest that horizontal differences in the occupational structure can be important predictors of workforce participation after retirement and should be taken into account in future studies.

It is also worth looking at occupational prestige in relation to wealth since the interplay of these two factors differs for the groups with lowest and higher retirement benefits. One potential way to extend this study is to analyse the motivations which drive respondents with low pre-retirement occupational prestige to continue working after retirement. There is a possibility that respondents working in low-prestige occupations before retirement are subsequently driven by purely financial motivations, yet the example of farm owners shows that it is not necessarily the only factor. Similarly, it would be worth giving more thorough consideration to the motivations of people who work after retirement despite receiving quite high retirement benefits. However, if we want to take into account various psychological constructs such as people’s motivation to continue working after retirement, we are faced with an additional constraint. In typical social surveys, collecting data on psychological constructs is more difficult than collecting opinions or factual data. The same problem might emerge if we want to assess other psychological aspects of attachment to the job, e.g., job satisfaction.

This paper contributes to the field of studies on the relationship between SES and workforce participation after retirement in line with the cumulative advantage/disadvantage theory and shows that resources accumulated by individuals during the life course can determine their chances of working after retirement, much like individual motivations or organisational characteristics do. On the other hand, our results remind us to be cautious: what is an advantage for some, can be a disadvantage for others. High-level professionals probably treat their work after retirement as an advantage, while for people who had been employed in low-prestige occupations and received low retirement benefits working after retirement is just a necessity. It seems that the most important good is the degree of choice that individuals have. This degree is a consequence, among others, of the life course history and one’s previous position in the social structure. The ways of accumulating immaterial goods such as choice are, however, not always linear. Ferraro and Shippee (Ferraro and Shippee 2009) who took the cumulative advantage/disadvantage theory as a starting point for developing their own cumulative inequality theory, highlight the complexity of links between different stages of life.

Apart from its theoretical contribution to literature, this paper can serve as a source of recommendations for the policy makers responsible for the labour market activity of older Poles. The results of our analysis suggest that policy makers should not concentrate only on the issue of the ageing society but also on inequalities of resources, which have an impact on citizens’ chances and motives related to workforce participation in older age.

There are certain limitations of the study that need to be mentioned here. Firstly, in order to assess the wealth of retirees we used only the variable referring to the amount of retirement benefits. The unavailability of comprehensive information on all sources of retirees’ income may weaken the strength of our conclusions. Due to incomplete data, we did not take into account that many older people in Poland have loans to repay.^14^ Secondly, we were not able to fully use the great potential of the panel data in this paper to study workforce participation of the retirees due to some methodological shortcomings, e.g. the fact that some questions that, in our view, are important predictors of employment after retirement were not asked to all the respondents in all the waves. We assume that panel studies of aging respondents would be continued in the subsequent waves of the study, enabling a more accurate analysis with respect to changes occurring over time.

## Appendix 1

**Table 5:** Matrix of correlations

		Working after retirement	Education (secondary and above)	Occupational prestige of the last job	Low retirement benefits	Male	Age in years	Married or cohabiting	Poor physical ability NHP	Self-assessment of health	Farmer
Working after retirement	Person’s *r*	1	0.100^**^	0.131^**^	0.023	0.044	−0.223^**^	0.070	−0.163^**^	0.126^**^	0.013
	N	787	786	773	753	787	787	787	787	755	772
Education (secondary and above)	Pearson’s *r*	0.100^**^	1	0.710^**^	−0.271^**^	−0.026	−0.099^**^	0.114^**^	−0.208^**^	0.167^**^	−0.293^**^
	N	786	786	772	752	786	786	786	786	754	771
Occupational prestige of the last job	Pearson’s *r*	0.131^**^	0.710^**^	1	−0.310^**^	0.055	−0.089^*^	0.130^**^	−0.229^**^	0.165^**^	−0.356^**^
	N	773	772	773	740	773	773	773	773	743	772
Low retirement benefits	Pearson’s *r*	0.023	−0.271^**^	−0.310^**^	1	−0.178^**^	−0.069	−0.003	0.147^**^	−0.201^**^	0.453^**^
	N	753	752	740	753	753	753	753	753	723	739
Male	Pearson’s *r*	0.044	−0.026	0.055	−0.178^**^	1	0.030	0.300^**^	−0.200^**^	0.093^*^	−0.071^*^
	N	787	786	773	753	787	787	787	787	755	772
Age in years	Pearson’s *r*	−0.223^**^	−.099^**^	−0.089^*^	−0.069	0.030	1	−0.269	0.352^**^	0.034	0.120^**^
	N	787	786	773	753	787	787	787	787	755	772
Married or cohabiting	Pearson’s *r*	0.070	0.114^**^	0.130^**^	−0.003	0.300^**^	−0.269^**^	1	−0.202^**^	0.023	−0.098^**^
	N	787	786	773	753	787	787	787	787	755	772
Poor physical ability NHP	Pearson’s *r*	−0.163^**^	−0.208^**^	−0.229^**^	0.147^**^	−0.200^**^	0.352^**^	−0.202^**^	1	−0.305^**^	0.161^**^
	N	787	786	773	753	787	787	787	787	755	772
Self-assessment of health	Pearson’s *r*	0.126^**^	0.167^**^	0.165^**^	−0.201^**^	0.093^*^	0.034	0.023	−0.305^**^	1	−0.152^**^
	N	755	754	743	723	755	755	755	755	755	742
Farmer	Pearson’s *r*	0.013	−0.293^**^	−0.356^**^	0.453^**^	−0.071^*^	0.120^**^	−0.098^**^	0.161^**^	−0.152^**^	1
	N	772	771	772	739	772	772	772	772	742	772

* p < 0.05, ** p < 0.01

*Source: POLPAN 2013*

## Footnotes

1 By social benefits, the authors of the study understand mainly many different kinds of old-age pensions, pre-retirement benefits or allowances, or disability pensions. They do not take into account unemployment benefits, family benefits, care benefits, rehabilitation or sickness allowances, or compensation benefits other than those intended for teachers.

2 The required history of paying pension contributions is slightly different in such a case. Currently (in 2017), it is 22 years for women and 25 years for men.

3 For the sake of simplicity, we disregard the population working in uniformed services (military, police etc.).

4
http://www.spoleczenstwoobywatelskie.gov.pl/dyskryminacja-ze-wzgledu-na-wiek

5 OECD defines net pension replacement rate as the individual net pension entitlement divided by net pre-retirement earnings, taking into account personal income taxes and social security contributions paid by workers and pensioners.

6 It is worth mentioning that there is no simple linear relation between the financial status of retirees and the occupational prestige of their pre-retirement occupation.

7 According to the data published by the Polish Agency for Restructuring and Modernisation of Agriculture (ARMA); www.arimr.gov.pl.

8 The sixth wave of the POLPAN study was funded by the Polish National Science Centre, under a grant number 2011/02/A/HS6/00238.

9 We have excluded two cases where respondents were working and receiving a relatively high salary and, at the same time, they were receiving a reduced amount of retirement benefits. We suppose that the former caused the latter.

10 The result is consistent with the findings obtained in the survey conducted by the Polish Public Opinion Research Center (CBOS 2012) in 2012, when 11% of retirees declared that they were working for pay.

11 This scale is very similar to the Standard International Occupational Prestige Scale, developed by Donald J. Treiman (Treiman 1977).

12 In our subsample, Pearson’s r equals 0.86 for the correlation with the 1979 Scale of Socio-Economic Status (SES) and 0.93 for the scale of skill requirements (Domański et al. 2009). This means that the scale of occupational prestige used in this study adequately describes the social position of an individual in the social structure.

13 The correlation table of all variables used in the study can be found in Appendix Table 5.

14 In POLPAN 2013, several versions of the questionnaire were used, and not all of them contained a question on loans and credits.
